# Natural Compounds for the Prevention and Treatment of Cardiovascular and Neurodegenerative Diseases

**DOI:** 10.3390/foods10010029

**Published:** 2020-12-24

**Authors:** Rosalba Leuci, Leonardo Brunetti, Viviana Poliseno, Antonio Laghezza, Fulvio Loiodice, Paolo Tortorella, Luca Piemontese

**Affiliations:** Dipartimento di Farmacia-Scienze del Farmaco-University of Bari “Aldo Moro”, Via E. Orabona 4, 70125 Bari, Italy; r.leuci6@studenti.uniba.it (R.L.); leonardo.brunetti@uniba.it (L.B.); v.poliseno1@studenti.uniba.it (V.P.); antonio.laghezza@uniba.it (A.L.); fulvio.loiodice@uniba.it (F.L.); paolo.tortorella@uniba.it (P.T.)

**Keywords:** secondary metabolites, plants, fungi, food supplements, cardiovascular diseases, neurodegenerative diseases, Alzheimer’s disease, metabolic syndrome

## Abstract

Secondary metabolites from plants and fungi are stimulating growing interest in consumers and, consequently, in the food and supplement industries. The beneficial effects of these natural compounds are being thoroughly studied and there are frequent updates about the biological activities of old and new molecules isolated from plants and fungi. In this article, we present a review of the most recent literature regarding the recent discovery of secondary metabolites through isolation and structural elucidation, as well as the in vitro and/or in vivo evaluation of their biological effects. In particular, the possibility of using these bioactive molecules in the prevention and/or treatment of widely spread pathologies such as cardiovascular and neurodegenerative diseases is discussed.

## 1. Introduction

Fungi and plants represent an important source of numerous bioactive compounds and have historically been used for medicinal purposes by virtually all human cultures [1].

Plants produce various secondary metabolites (SMs) in order to defend themselves from external attacks and as signals. These SMs show interesting biological and pharmacological activities: for this reason, they are often isolated and used for therapeutic purposes [2]. Plant-derived compounds are currently used in oncology therapy worldwide because they are considered less toxic and thus better accepted by patients [3], even if this consideration cannot be extended to all natural compounds and can be dangerous. Taxanes, used for the treatment of patients with breast cancer, are an excellent example of a valuable drug: paclitaxel is isolated from the bark of *Taxus brevifolia* (Pacific yew tree), while docetaxel is extracted from the needles of *Taxus baccata* (European yew tree) [4]. Other anticancer treatments obtained from plants are the vinca alkaloids vincristine and vinblastine, derived from the periwinkle plant *Catharanthus roseus* [5].

Polyphenols are another remarkable class of plant-derived SM, endowed with protective effects against pathologies such as cancer, cardiovascular diseases, diabetes, and neurodegenerative disorders [6]. These are classified into phenolic acids, flavonoids, stilbenes, coumarins, lignins, and tannins. Coumarins are found in a variety of plants such as tonka bean (*Dipteryx odorata*), sweet woodruff (*Galium odoratum*), sweet grass (*Hierochloe odorata*), deer-tongue (*Dichanthelium clandestinum*), vanilla grass (*Anthoxanthum odoratum*), mullein (*Verbascum* spp.), and sweet-clover (*Melilotus* sp.) [7]. Resveratrol, a stilbenoid present in many fresh fruits and plants such as *Polygonum cuspidatum*, *Arachis hypogea, Cassia* sp., *Eucalyptus*, *Morus rubra*, and *Vitis vinifera*, has been reported to have numerous biological properties, such as antioxidant, anti-inflammatory, anti-cancer, anti-aging, anti-obesity, anti-diabetes, cardioprotective and neuroprotective effects [8].

In recent years, the interest on the medicinal properties of compounds from *Cannabis* species has been steadily growing. More than 100 phyto-cannabinoids have been identified from *C. sativa*; among them, the most potent psychoactive activity is displayed by *trans*-Δ^9^-tetrahydrocannabinol (THC). According to several studies, cannabis derivatives can be useful in conditions such as pain, anorexia-cachexia, skin pathologies, neurodegenerative diseases, epilepsy, sleep disorders and infections. However, legislation regarding these compounds is still ambiguous, insufficient, and plagued by controversies linked to their adverse effects and their consumption as recreational drugs [9,10].

Fungi also produce bioactive natural products that are exploited for pharmaceutical purposes. Fungal metabolites with clinical use include beta lactams, e.g., penicillins G and V, statins, cholesterol-lowering blockbuster drugs, the immunosuppressant cyclosporin and the anti-migraine ergotamine [11]. Beta-lactams are the most widely used class of antibiotics that, with the discovery of penicillin, produced by the fungus *Penicillium notatum*, early in the twentieth century, marked a new era for the treatment of bacterial infections [12]. Cyclosporin is employed for the treatment of autoimmune diseases such as psoriasis; it is a peptide isolated from *Tolypocladium inflatum* [13].

Many natural compounds from fungi and plants are extensively used as food supplements for the treatment and prevention of neurodegenerative and cardiovascular diseases [14], showing the growing interest in this field of research. Among the best-selling products, monacolin K, a component of red yeast rice fermented with several patented *Monascus purpureus* strains, is a widely discussed case. Considering its chemical structure and biological activity [15], the use of the food supplement containing this bioactive compound should be more strictly regulated. This review focuses on another important aspect of research regarding natural compounds: the isolation of secondary metabolites of fungi and plants and their biological evaluation as potential useful compounds for neurodegenerative and cardiovascular disorders.

## 2. Natural Compounds and Neurodegenerative Diseases

The prevention and treatment of neurodegenerative diseases (NDs), such as Parkinson’s disease (PD) and Alzheimer’s disease (AD), is an important avenue of research due to the increasing occurrence of these pathologies in the rapidly aging world population. Their multifactorial nature complicates their diagnostic and therapeutic profile and only few drugs are available [16,17,18]. Lifestyle factors, including dietary habits, influence the development of NDs, further cementing the role of food-derived compounds such as plant SMs in the long-term physiological balance of the nervous system [19]. Table 1 summarizes the body of literature regarding plant and fungal SMs with potential activity toward NDs.

Oxidative stress and neuroinflammation are considered two of the causes of NDs [19,36]. For this reason, neuroprotective agents targeting these pathological factors can be useful for the prevention and treatment of these disorders [36]. In recent years, several natural compounds have been explored for their neuroprotective and antioxidant activity. For example, polyphenols, which are secondary metabolites of plants present in various food and drinks, have shown important antioxidant properties [37]. However, numerous studies in the recent past have been focused on different and more original matrices.

*Cetraria islandica* L. *Ach* (or Iceland moss), as an example, is the most famous cetrarioid lichen species. It has been used for the treatment of various inflammatory diseases, and recently the neuroprotective properties of its major metabolite, fumarprotocetraric acid (FUM) were evaluated on neuron-like SH-SY5Y cells and glial U373-MG cells. FUM revealed different activities, acting as a scavenger of peroxyl radicals, decreasing reactive oxygen species (ROS) production, reducing GSH depletion and increasing the ratio of reduced glutathioneto oxidized glutathione (GSH/GSSG ratio). Moreover, FUM decreased mitochondrial Ca^2+^ levels, protected the mitochondrial membrane against H_2_O_2_-induced damage, suppressed H_2_O_2_-induced expression of protease caspase-3, decreased pro-apoptotic factor Bax levels and increased the anti-apoptotic Bcl-2 levels [20].

Another interesting research is the in vitro screening of the antioxidant action of six diterpene derivatives, named Gracilin A, H, K, J, L, and tetrahydroaplysulphurin-1, isolated from *Spongionella* sp., a marine sponge. The two parameters for the evaluation of antioxidant activity were MTT (3-(4,5-dimethylthiazol-2-yl)-2,5-diphenyltetrazolium bromide) assay and LDH levels: the first is correlated with mitochondrial function, the second is a cytoplasmic enzyme released in the culture medium following cell membrane damage. The tested compounds showed significant neuroprotective activity, interacting with targets such as mitochondrial oxidative phosphorylation and kinases involved in apoptosis [21].

Looking at innovative targets, a recent paper describes Macamides, a group of secondary metabolites isolated from the plant *Lepidium meyenii* (Maca). These compounds are benzylamides of fatty acids, active as analogues of the endocannabinoid anandamide (AEA) and studies have demonstrated that they inhibit fatty acid amide hydrolase (FAAH), blocking AEA hydrolysis. Gugnani et al. demonstrated a neuroprotective role of macamides in vitro and in vivo. Macamides reduced Mn-induced mitochondrial toxicity in glioblastoma U-87 MG cells, probably by binding the CB_1_ receptor, and it could thus be useful in the treatment of neurodegenerative diseases, especially Alzheimer’s Disease. Like AEA, macamides can interact with PPARγ, regulating inflammation, energetic metabolism and glucose homeostasis, all important factors for the prevention of AD [22,38].

Other interesting bioactive secondary metabolites are butenolides, from the fungus *Aspergillus terreus*. The chemical structures of these compounds were recently elucidated and their effects against the expression of TNFα in lipopolysaccharide (LPS)-activated BV2 microglia cells were tested. The most promising compound was asperteretal F, which inhibited the expression of TNFα in a dose-dependent mode, making it an anti-neuroinflammatory candidate for the treatment of NDs [23].

Sarcophytolide, instead, is a lacton cembrane diterpene derived from soft coral *Sarcophyton glaucum* that was recently shown to possess antimicrobic activity towards *Staphylococcus aureus*, *Pseudomonas aeruginosa*, and *Saccharomyces cerevisiae*. Moreover, pretreatment of primary cortical cells with sarcophytolide had a strong cytoprotective effect against glutamate-induced neurotoxicity and increased the expression of Bcl-2. This mechanism confirmed by evidence that sarcophytolide showed a cytoprotective activity only if added before and not after the exposure of neuronal cells to glutamate [24].

Meanwhile, Rupcic et al. discovered two new metabolites of the medicinal mushrooms *Hericius erinaceus* and *Hericius flagellum* (a rare species). They determined the chemical structures of these new compounds and of other metabolites previously isolated from the two species through Nuclear Magnetic Resonance (NMR) and High-Resolution Mass Spectroscopy (HRMS), identifying them as cyathane diterpenes. All these compounds were tested in vitro on PC12 cells for their neurotrophic activity, showing that, although none of them was endowed with intrinsic neurotrophic properties, all of them promoted the production of neurotrophins NGF (Nerve Growth Factor) and BDNF (Brain-Derived Neurotrophic Factor) in astrocytic cells [25].

### 2.1. Alzheimer’s Disease

Alzheimer’s disease (AD) is the most common neurodegenerative disorder, characterized by several cognitive and behavioral disfunctions, and it affects mainly old people, although rare early onset of this disease is also known [39,40]. Several hypotheses have been formulated regarding its pathogenesis, including deposition of amyloid β (Aβ), damaged cholinergic transmission, oxidative stress and hyperphosphorylated Tau aggregation [41,42].

According to the so-called “cholinergic hypothesis”, AD is linked to decreased levels of acetylcholine (ACh) accompanied by a loss of cholinergic neurons in the central nervous system. In order to raise the concentration of this neurotransmitter, inhibitors of acetylcholine esterase (AChE), the main enzyme responsible for the degradation of ACh, have found use in clinical practice, improving functional autonomy and cognitive functions in AD patients [43]. Among these drugs are natural compounds such as galantamine, the most important *Amaryllidaceae* alkaloid [44]. For this reason, the potential use in AD of other *Amaryllidaceae* alkaloids, isolated from *Narcissus tazetta* L., was recently evaluated. Their structures were determined by NMR and mass spectroscopy, while in vıtro AChE and butyrylcholinesterase (BChE) inhibitory activity was evaluated using Ellman’s colorimetric method, with galantamine as reference. (+)-11-Hydroxygalanthine had the highest selective inhibitory activity on AChE, and narcissidine also inhibited AChE rather than BChE; while (−)-pancratinine-C was a selective BChE inhibitor. Docking studies confirmed bioactivity results [26].

The screening of libraries of natural compounds has also proven to be a promising strategy to identify multi-target compounds for the treatment of AD. Embelin, a 1,4-benzoquinone isolated from *Embelia ribes* fruits, recently emerged from one such screening, showing inhibitory activity towards AChE, BChE and beta-secretase 1 (BACE-1, involved in the deposition of Aβ). Another two natural products present in the library, L-tetrahydropalmatine and papaverine, exhibited a good inhibition of AChE. Moreover, embelin, in LS-180 cells, acted as an inductor of P-gp, an ATP-dependent efflux pump situated in the blood-brain barrier (BBB) whose decreased levels can lead to the accumulation of Aβ plaques [27].

Anti-AChE activity was exhibited also by four secondary metabolites (helminthosporin, emodin, chrysophanol, and physcion) of the African medicinal plant *Rumex Abyssinias*. These compounds, sharing an anthraquinone structure, were isolated and showed significant AChE inhibitory activity. Helminthosporin also displayed activity as a non-competitive BChE inhibitor, while the other compounds were only weakly active against this target. Moreover, the tricyclic flat structure makes helminthosporin lipophilic enough to cross the BBB, balancing its low LogP value [28].

Biologically active secondary metabolites such as phenols, alkaloids flavonoids, terpenes, sterols, and tannins from the ethanolic extract of *Oxalis corniculata* L. are endowed with carbonic anhydrase and cholinesterase inhibitory activity, with potential uses against epilepsy and Alzheimer’s disease, respectively. Moreover, nine flavonoids were isolated from chloroform and ethyl acetate fractions, displaying ChEs and carbonic anhydrase II (CA-II) inhibitory activity [29].

Lichens have also been used as a source of natural compounds for the treatment of AD. During a recent study conducted on murine neuroblastoma Neuro2A cells, lichen-derived secondary metabolites (atranorin, perlatolic acid, physodic acid and usnic acid) displayed an important increase in neurite outgrowth, with perlatolic acid achieving better results than reference compound resveratrol. MTT assays revealed that only usnic acid showed cytotoxicity at neurotrophic doses. The molecular mediators of these effects are NGF, which is upregulated by atranorin, perlatolic acid and physodic acid, and BDNF, which is upregulated by atranorin and physodic acid. Only perlatolic acid had a potential inhibitory activity on AChE, at a concentration that was comparable with galantamine and lower than biroquinone, another lichen metabolite reported as AChE inhibitor [30].

Detailed knowledge of the structure of plant and fungal secondary metabolites has also proven useful directing screening activities. Due to their similarity to existing nuclei used in AChE inhibitors and metal chelators, fungal secondary metabolites tenuazonic acid (TA), *epi*-radicinol (ROH), mycophenolic acid (MA), radicinin (RAD), visoltricin/fungerin (FU) and plant metabolite 6-methoxymellein (6-MM) were screened for various activities such as inhibition of AChE, BChE and Aβ-aggregation, antioxidant effect and Cu and Zn interaction. A preliminary UV spectrophotometry test for metal chelation of Cu (II) and Zn (II) at physiological pH revealed that TA, MA and 6-MM probably interacted with Cu (II). TA and ROH exhibited a significant selective AChE inhibitory activity, while FU was the only compound that inhibited BChE. 2,2-diphenyl-1-picrylhydrazyl (DPPH) radical scavenging activity assay suggested that TA and MA behaved as antioxidants. Moreover, all molecules were inhibitors of Aβ_1–40_ aggregation, as demonstrated by spectrofluorimetric assays, with ROH being the most active compound [31].

### 2.2. Parkinson’s Disease

Parkinson’s disease (PD) is a very common neurodegenerative disease characterized by motor symptoms, such as rigidity, bradykinesia and tremor, and non-motor symptoms, including sleep disorders and cognitive abnormalities [45]. The principal causes of this pathology are the depletion of dopamine in nerve terminals to the striatum and the progressive degeneration of dopaminergic neurons in the substantia nigra [46]. The therapy involves the use of levodopa associated with an inhibitor of the peripheral metabolism of levodopa, for example carbidopa. Additionally, other drugs can be administrated, such as dopaminergic agonists, monoamine oxidase-B inhibitors and catechol *O*-methyltransferase inhibitors [47].

Monoamine oxidases (MAOs) are enzymes responsible for the oxidative deamination of both xenobiotic and endogenous neurotransmitters. Both MAO isoforms (MAO-A and MAO-B) are involved in the degradation of dopamine; however, MAO-B is the predominant isoform in the human brain. For this reason, MAO-B selective inhibitors, like selegiline and rasagiline, are used for the treatment of PD, raising striatal dopaminergic activity through the inhibition of dopamine metabolism [48,49,50].

In recent years, many natural inhibitors of MAO-B have been discovered and characterized, representing a powerful source of inspiration for further drug development [51].

MAO-A and MAO-B inhibitory activities of seven compounds isolated from the extract of *S.flavescens* were recently investigated, highlighting (−)-maackian as a potent inhibitor of MAO-B and as a candidate for the development of drugs for Parkinson’s disease. The structurally related compound (−)-4-hydroxy-3-methoxy-8,9-methylenedioxypterocarpan was a non-selective inhibitor of both isoforms, as well as formononetin and genistein. Sophora-flavanone B weakly inhibited MAO-A, but not MAO-B, while kushenol F showed a good inhibitory activity on MAO-A and a weak one on MAO-B [32].

A dichloromethane extract of *Renealmia Alpinia*, was recently shown to possess a potent inhibitory activity towards both MAO-A and MAO-B. From this extract, desmethoxyangonin, a kavalactone, was isolated. It exhibited a potent, selective and competitive inhibition of MAO-B rather than MAO-A, confirmed by molecular modeling studies, leading to its selection as a candidate for further drug development. Other isolated molecules from the extract displayed moderate inhibition of both isoforms [33].

The aggregation of α-synuclein (αSN) in cytoplasmic inclusions called Lewy bodies is another important pathogenetic mechanism involved in PD. The formation of αSN fibrils leads to the disruption of synaptic homeostasis and neurodegeneration. While the exact mechanism is not clear, αSN is an interesting target for natural and synthetic drugs alike [52,53].

A natural compound extracted from *Ginkgo biloba* leaves, ginkgolic acid (GA), and its related molecule anacardic acid (AA) were screened for their influence on αSN aggregates. The treatment of KCl-depolarized SH-SY5Y neuroblastoma cells with GA and AA led to a progressive and relevant decrease in αSN-positive aggregates, probably also due to increased activation of macro-autophagy, that resulted in increased survival of the neural cells [34]. It should be noted that extracts from this plant, containing as low as 5 mg/kg GA, still showed potential to improve cognitive function in mild dementia patients after >24 weeks administration at a dosage of 240 mg/day [54]. Dihydromyricetin (DHM) is a flavonoid isolated from *Ampelopsis grossedentata*, a herb used in traditional Chinese medicine. Previous studies demonstrated that this compound played a neuroprotective role, and DHM, as proved by MTT assays on PC12 cells, blocked αSN fibrillo-genesis and its cytotoxicity. Moreover, DHM also disassembled preformed αSN fibrils, making it a potential molecule for the therapy of PD [35].

## 3. Metabolic Syndrome and Cardiovascular Risk

Metabolic syndrome (MS) is an increasingly prevalent condition that comprises a variety of pathological states, ranging from type 2 diabetes mellitus (T2DM) to obesity, hyperlipidemia and hypertension [55]. Obesity and hyperlipidemia are known causes of hyperinflammatory states, leading to the expression of pro-inflammatory cytokines and to reduced levels of nitric oxide (NO, which regulates endothelial homeostasis). Moreover, insulin-resistance leads to a blocking of the vasodilating effects of insulin itself, causing endothelial dysfunction. All these factors increase cardiovascular risk [56] and contribute significantly to the morbidity and mortality of cardiovascular diseases (CVD), the most prevalent cause of death worldwide.

Unfortunately, available drugs for the treatment of metabolic disorders are few and sometimes expensive: for this reason, researchers are focusing on the discovery of new and effective drugs [57,58]. In the last decade, it has become clear that an approach covering all underlying pathological conditions is required for the therapy of metabolic syndrome. Although the first step in such a therapeutic regime consists of increased physical exercise and dietary intervention, in many cases pharmacological action is necessary [59].

Over the years, the different aspects of metabolic syndrome have been thoroughly studied, and a number of potential molecular targets have been identified and characterized. Peroxisome Proliferator-Activated Receptors (PPARs), a family of nuclear receptors involved in all aspects of energetic metabolism, have attracted much interest as targets for therapy, considering their important role in the recent past in the treatment of both dyslipidemic and glucose-related pathologies [60,61]. Three PPAR receptor subtypes have been identified to date, respectively PPARα, PPARγ and PPARδ, each of which binds different endogenous ligands and is selectively expressed in different organs and tissues [62,63,64]. Their differential expression and ligand specificity mirror their different physiological roles: PPARα and PPARδ are mostly responsible for catabolic functions such as lipid oxygenation and glucose consumption, while PPARγ regulates glucose uptake and anabolic functions, particularly lipid storage and adipose tissue formation [65].

An enhanced understanding of the pharmacological profile of PPAR agonists has led to increased interest in their development, focusing particularly on obtaining a selective modulation of their subtypes, in order to maximize their beneficial effects and to minimize adverse reactions [66,67,68,69]. This focus on PPAR agonists is, however, not exclusive to medicinal chemists, and PPAR agonism or related activities have been reported for various secondary metabolites derived from plant and fungal extracts. 

Table 2 summarizes the body of literature regarding plant and fungal SMs with potential activity toward MS and CVD.

*Acalypha fluticosa* extracts were recently screened for PPAR agonism and anti-inflammatory properties. Following a preliminary screening, four compounds were isolated from the methanol extract, namely 2-methyl-5,7-dihydroxychromone-5-*O*-b-d-glucopyranosid, acalyphin, apigenin, and kaempferol-3-*O*-rutinoside. Moreover, an acetylated derivate of chromone glucoside was synthesized and tested. In vitro on human hepatoma (HepG2) cells, acalyphin exhibited a specific PPARγ agonist activity, while apigenin revealed a weak PPARα agonism. Chromone glucoside displayed activity as a dual PPARα/γ agonist, while its acetylated derivative showed increased activity towards PPARα. These molecules, in particular acalyphin, also had anti-inflammatory properties, probably due to the inhibition of NF-κB and/or iNOS. Importantly, tested extracts and compounds were not found to be cytotoxic in vitro against human cells [70].

The ethanolic extract of stems of the Panamanian plant *Talisia nervosa* was also studied, along with its isolated components, for potential use in metabolic disorders. The extract displayed dual PPARα/γ agonism and also increased liver X receptor (LXR) activation. Therefore, four secondary metabolites, namely (–)-catechin, methyl gallate, ethyl gallate, and β-d-glucopyranose-1,4,6-tris(3,4,5-trihydroxybenzoate) were isolated and characterized. In vitro assays on HepG2 revealed that while (–)-catechin activated only PPARγ and not PPARα, the other three compounds activated PPARα, PPARγ and LXR, with methyl gallate being more potent than ethyl gallate on all three targets. The two gallates also reduced nitric oxide (NO) production in mouse macrophages (RAW 264.7) cells [71].

Aside from five known bile acids, a new bile acid trifluoroacetate was isolated from jellyfish-derived fungus *Penicillium chrysogenum* J08NF-4. Its chemical structure and mechanism of action were similar to those of synthetic steroid mifepristone, which is clinically used for the treatment of hypercholesterolemia and recently turned out to be a PPARγ agonist. Docking studies confirmed that this new bile acid, like mifepristone, is capable of binding the ligand binding domain (LBD) of PPARγ, thus suppressing the NF-kB pathway and downregulating the pro-inflammatory mediators iNOS, TNF-α, and NO, with generalized anti-inflammatory effects, as confirmed by in vitro assay on LPS-induced RAW 264.7 macrophages [72].

Other than plants and fungi, marine cyanobacteria have been a promising source of secondary metabolites with potential medicinal use. As an example, chlorophyll derivatives 13^2^-hydroxy-pheopytin-a and the novel 13^2^-hydroxy-pheofarnesin-a were recently isolated from *Cyanobium* sp. LEGE 07,175 -and *Nodosilinea* sp. LEGE 06001, respectively. The zebrafish Nile red fat metabolism assay confirmed a neutral lipid-reducing activity of both compounds after 48 h of exposure, with no toxic effects. In order to explain the biological mechanism behind this activity profile, 13^2^-hydroxy-pheopytin-a was found to increase PPARγ mRNA expression. In light of these data, the presence of this compound in several foods, such as spinach, cabbage, *Spirulina* and *Chlorella*, makes it an important nutraceutical agent [73].

### 3.1. Diabetes

Type 2 diabetes (T2DM, or non-insulin dependent diabetes mellitus) is the most common form of diabetes. It is characterized by peripheric insulin resistance and hyperglycemia, leading to a loss of pancreatic β-cells which further exacerbates the pathology. No cure is currently available for this disease, however, various drugs capable of ameliorating the patients’ quality of life and useful to control the disease have been approved [89]. Studies confirmed that natural products can also be used for the prevention and/or treatment of type 2 diabetes [90].

The therapy of T2DM usually focuses on reducing postprandial glycemia, restoring peripheric insulin sensitivity and improving β-cell survival. Other than the previously discussed PPARγ, molecular targets for the therapy of T2DM include intestinal α-glucosidase and pancreatic α-amylase [91], which catalyze the hydrolysis of dietary carbohydrates and whose inhibition leads to decrease of postprandial blood glucose levels, thanks to a decreased digestion and uptake of carbohydrates [86]. Dipeptidyl peptidase-4 (DPP-4) is another target for the treatment of this pathology: this enzyme metabolizes glucagon-like peptide-1 (GLP-1), an incretin hormone which stimulates insulin secretion, inhibits glucagon secretion and delays gastric emptying [92]. Moreover, recent studies have proven the role of enzyme protein tyrosine phosphatase 1B (PTP1B) in the negative regulation of insulin signaling, making it a novel target for the treatment of T2DM [93].

An acidic polysaccharide from *Schisandra chinensis* (SCAP) was recently evaluated as a potential therapeutic agent in the streptozotocin-induced mouse model of diabetes. SCAP led to increased levels of fasting blood insulin and superoxide dismutase and to decreased levels of fasting blood glucose and malondialdehyde. This polysaccharide also prevented apoptosis of pancreatic β-cells through the up-regulation of factors such as BAX and Bcl-2, suggesting a possible mechanism for its antidiabetic activity. [75].

In another recent study, fourteen known compounds were isolated from *S. grandiflora* crude extract and tested against α-amylase and α-glucosidase in vitro. Two flavonoids (quercetin and kaempferol) and two terpenoids (vomifoliol and loliolide) showed inhibitory activity toward these enzymes, which was justified through docking studies. Finally, all bioactive molecules acted as antioxidants in the ABTS (2,2′-azino-bis(3-ethylbenzothiazoline-6-sulfonic acid) radical scavenging assay. Quantitative analysis highlighted high concentrations of the most active compounds in the plant extract, suggesting a potential use of the edible *S. grandiflora* for the control of postprandial blood glucose in diabetic patients [76].

Similarly, flavonoids kaempferol-3-*O*-rhamnoside and kaempferol, isolated from *Cassia bakeriana* (pink cassia) extracts, were also recently studied for their antidiabetic activities. While kaempferol was unambiguously able to inhibit α-amylase, the evaluation of the antioxidant activities of these compounds led to somewhat contrasting results, with both being active when tested via oxygen radical absorbance capacity (ORAC) method, and only kaempferol-3-*O*-rhamnoside being active in DPPH assay. It is worth noting that the isolated compounds are less active than the whole extract, suggesting the importance of synergic effects between the various components of the extract [77].

The leaf infusion of *Ocimum campechianum* (or wild basil) was reported to play an antidiabetic role through α-glucosidase inhibition. Five poly-methoxylated flavones were isolated and their structures were elucidated, with two of these compounds (methyl rosmarinate and rosmarinic acid) displaying strong inhibition of α-glucosidase in vitro and a marked decrease of blood glucose in vivo [78].

Natural compound stella-steroid was instead isolated from fungal plant pathogen *Ganoderma australe*, and its structure was elucidated via NMR spectroscopy. This compound showed inhibitory activity towards DPP-4 and α-glucosidase in silico and in vitro, with higher activity toward the latter target [80].

Two natural compounds from *Aspergillus terreus*, known as butyrolactone I and II, along with three synthetic acetylated derivatives of butyrolactone I, were also screened in a different study for their activity as α-glucosidase inhibitors and as antioxidants. Butyrolactone I showed both α-glucosidase inhibitory and antioxidant activities, while butyrolactone II was the most potent antioxidant compound, but had a lower inhibitory activity toward α-glucosidase. Synthetic derivatives were less active, probably because of the acetylation of the hydroxyl groups of butyrolactone I [79].

Another fungus from the *Aspergillus* genus, *Aspergillus sydowii*, was found to produce a compound with significant antidiabetic potential, asperentin B. In vitro, this compound inhibited PTP1B six times more strongly than the positive control suramin. Interestingly, the structurally related compound asperentin did not display any inhibitory activity towards PTP1B. Future studies on structure-activity relationships and chemical modifications will be necessary to explain and enhance the antidiabetic activity of asperentin B [81].

### 3.2. Obesity

Obesity and overweight, defined as excess body weight, affect a growing number of adults, children and adolescents. Obesity in particular is often associated with the development of other disorders such as T2DM, cardiovascular disease and nonalcoholic fatty liver disease. Behavioral interventions, including dietary changes and increased physical exercise, are important to prevent obesity and to induce weight loss, however pharmacological treatment is often necessary in obese patients [94,95,96].

A viable strategy for the treatment of metabolic disorders like obesity is decreasing the absorption of dietary components such as fats and carbohydrates through the inhibition of metabolic enzymes, including lipase and, again, α-glucosidase [97]. On the other hand, it is also necessary to act on signaling pathways that might be dysregulated as a result of obesity. Leptin is a protein involved in one such pathway; it is expressed in the adipocytes and it controls body weight and the mass of adipose tissue through the inhibition of food intake and the stimulation of energy expenditure. Defects in leptin production cause severe obesity [98].

A growing body of literature demonstrates the potential anti-obesity action of natural bioactive compounds, mostly derived from plants [99]. As an example, extracts from seeds and leaves *Moringa oleifera* were recently tested for various properties such as antioxidant, anti-inflammatory and anti-hyperlipidemic effects. Two sulfur-containing compounds were isolated from these extracts and were evaluated for their anti-adipogenic activity on pre-adipocyte 3T3-L1 cell line. One compound had no significant anti-adipogenic activity, while the other one showed a significant inhibition of intracellular lipid accumulation, probably due to the presence of an isothiocyanate group (ITC) in its structure. For this reason, a series of ITC derivatives were prepared, and most of them also showed anti-adipogenic activity [82]. Similarly, three eugenol diglycosides and three β-carboline alkaloids isolated from Garlic (*Allium sativum* L.) were screened on 3T3-L1 cells for their effects on adipogenesis and lipid metabolism. Among them, one β-carboline alkaloid inhibited adipogenesis and lipid accumulation through the regulation of adipogenic, lipogenic and lipolytic genes [83].

In another recent study, two natural labdane diterpenes and a drimane sesquiterpene were isolated from the hexane extract of *Curcuma amada* and they were tested for their inhibitory activity against rat intestinal α-glucosidase and porcine pancreatic lipase. One of the two diterpenes showed relevant inhibitory activity toward both enzymes. Therefore, some semi-synthetic derivatives of this molecule were prepared and screened: a reduced derivative maintained α-glucosidase inhibitory activity, while it lost lipase inhibition. At the opposite end of the spectrum, oxidized and acetate derivatives acted as good lipase inhibitors but had a weak α-glucosidase inhibitory activity [84].

Another terpene, specifically a pentacyclic triterpene from the roots of *Tripterygium Wilfordi*, was recently discovered to be a leptin sensitizer. In hyperleptinemic diet-induced obese (DIO) mice it led to effects such as a significant decrease of food intake, increased hypothalamic leptin sensitivity, and weight loss, a promising profile for the treatment of obesity [88].

### 3.3. Hypertension and Hyperinflammation-Managing Cardiovascular Risk in Metabolic Syndrome

Hypertension and hyperinflammation are another important component of MS which, along with hypercholesterolemia, cause atherosclerosis and therefore significantly raise the cardiovascular risk linked to this syndrome [55].

In this case, too, secondary metabolites of plants and fungi have shown promise as preventative or therapeutic agents. As an example, honokiol, a natural compound from magnolia plants, was very recently shown to ameliorate Angiotensin II-induced hypertension and endothelial dysfunction via inhibition of histone deacetylase 6 (HDAC6) [85].

A very promising way to prevent cardiovascular risk related to hyperinflammation and atherosclerosis is to target the macrophages that accumulate in the site of the atherogenic lesion and bind low density lipoprotein (LDL), transforming in foam cells and driving inflammation further [100]. An aqueous bark extract from the plant *Terminalia Arjuna*, containing a number of polyphenols including gallic acid, epigallocatechin gallate and ellagic acid, displayed significant activity in stimulating apoptosis in macrophages and foam cells during the early stages of atherogenesis, thereby driving back inflammation and potentially reducing cardiovascular risk [101]. Similar effects were shown by ginseng-derived compound ginsenoside K [86], while lupeol, a pentacyclic terpene which can be found in a variety of fruits and vegetables, including mango, red grapes and tomato, was shown to be capable of shunting macrophage development towards the anti-inflammatory, reparative M2 phenotype (as opposed to the pro-inflammatory M1 phenotype) [87].

The formation of advanced-end glycation products (AGEs) is involved in several pathologies such as atherosclerosis, arterial stiffness, but also Parkinson’s disease and Alzheimer’s disease. In a recent study, a group of lichen secondary metabolites and one semisynthetic derivative were shown to possess relevant inhibitory activity towards AGE formation. Although some of these compounds were endowed with antioxidant activity, it was not necessarily linked to the inhibition of AGE formation. Moreover, the tested compounds proved to have vasodilative effects, potentially useful in alleviating hypertension linked to atherosclerosis [74].

Finally, three new alkaloids (communesin I, fumoquinazoline Q and protuboxepin E) and nine known alkaloids (communesin A and B, cottoquinazoline A, prelapatin B, glyantrypine, protuboxepin A and B, chaetoglobosin C and penochalasin E) were recently isolated from the marine-derived fungus *Penicillium expansum* Y32. All molecules were screened in vivo on a zebrafish model for their cardiovascular effects. All of them exhibited a mitigative activity on bradycardia induced by astemizole; moreover, all compounds, except for communesin B, showed vasculo-genetic effects [58].

## 4. Conclusions

The research reviewed in this paper confirms the rising importance of natural compounds in the inspiration of new therapeutic protocols for the prevention and/or treatment of chronic diseases.

However, the use of secondary metabolites requires in many cases a specific formulation, when we consider that they are often insoluble in water, and the problems related to the abuse of supplements containing bioactive molecules, while outside the scope of this review, must still be carefully considered, taking into account the side effects of the active ingredients themselves and the possibility of external contamination [102]. The already mentioned case of Monacolin K, a blockbuster product which presents the same problems as statins (being a statin itself) and a high possibility of contamination with citrinin (fermented red rice with *Monascus Purpureus* strains is the only regulated matrix in EU for this mycotoxin [103]), must be a wake-up call to the food supplement industry. Therefore, future research must definitely focus on the analysis of natural contaminants such as ochratoxin A [104] and other mycotoxins, as well as residues of pesticides and heavy metals, if these bioactive compounds are to be included in plant food supplements [105].

The design of new potential drugs starting from the structures of natural compounds and the preparation of these molecules with a semi-synthetic or a total-synthetic approach will be another significant challenge in future years. Several research groups are working on this fascinating, though not always linear, route [72,82,106], and important results are expected.

## Figures and Tables

**Table 1:** Natural compounds and neurodegenerative diseases.

Source	Bioactive Compounds	Effects	Main Activities	Ref.
*Cetraria islandica* L. *Ach*	Furmarprotocetraric acid	Neuroprotective and antioxidant activities	Oxygen radical absorbance capacity (ORAC) 5.07 ± 0.43 μmol TE/mg	[20]
*Spongionella* sp.	Gracilin A, H, K, J, L, and tetrahydroaplysulphurin-1	Neuroprotective activity	(Caspase 3 inh.) 3.88–4.04 × 10^3^ RFU	[21]
*Lepidium meyenii*	*N*-(3-methoxybenzyl)oleamide, (*N*-(3-methoxybenzyl)linolenamide, *N*-(3-methoxybenzyl)linolenamide	Neuroprotective activity, peroxisome proliferator-activated receptor (PPAR) γ interaction, inhibition of fatty acid amide hydrolase (FAAH)	(PPARγ act., EC_50_) 20.4–22.6 μM	[22]
*Aspergillus terreus* Y10	Asperteretal F, G_1_, G_2_, H and others	Inhibition of Tumor Necrosis Factor α (TNFα)	(TNFα inh., IC_50_) 7.6 9.9 μM	[23]
*Sarcophyton glaucum*	Sarcophytolide	Antimicrobic and cytoprotective activities	(MIC) 0.13–0.22 μg/mL	[24]
*Hericius erinaceus* and *Hericius flagellum*	Erinacine A, B, C, E, F, and others	Neurotrophic activity	(increased NGF expression) 0.8–12 μg/mL	[25]
*Narcissus tazetta* L.	(−)-9-*O*-methylpseudolycorine, (−)-narcissidine, (−)-pancratinine-C, (+)-9-*O*-demethyl-2-a-hydroxyhomolycorine	Inhibition of acetylcholine esterase AChE and butyrylcholine esterase (BChE)	(AChE inh, IC_50_) 0.67–32.51 μM	[26]
*Embelia ribes* and others	Embelin and others	Inhibition of AChE, BChE and Beta-secretase 1 (BACE-1); induction of P-glycoprotein 1 (P-gp)	(AChE inh, IC_50_) 2.50–6.98 μM	[27]
*Rumex abyssinicus*	Helminthosporin, emodin, chryso-phanol, physcion	Inhibition of AChE and BChE	(AChE inh, IC_50_) 2.63–33.7 μM	[28]
*Oxalis corniculate* L.	Flavonoids 1-9	Inhibition of AChE, BChE and carbonic anhydrases II (CA-II)	(AChE inh, IC_50_) 49.52–109.55 μg/mL	[29]
Lichens	Atranorin, perlatolic acid, physodic acid, usnic acid and others	Neurotrophic activity and AChE inhibition	(AChE inh, IC_50_) 6.8–27.1 μM	[30]
Fungi and plants	Tenuazonic acid, *epi*-racidinol, mycophenolic acid, radicinin, visoltricin, 6-methoxymellein	Inhibition of AChE, BChE and Aβ-aggregation; antioxidant activity, metal chelation	(AChE inh, IC_50_) 6.86–11.4 μM	[31]
*S. flavescens*	(−)-maackian and others	Inhibition of monoamine oxidases (MAOs)	(MAO-B inh, IC_50_) 0.68–52.3 μM	[32]
*Renealmia Alpinia*	Desmethoxyangonin and others	Inhibition of MAOs	(MAO-B inh, Ki) 31–110 nM	[33]
*Ginkgo biloba*	Ginkgolic acid and anacardic acid	Decreased accumulation of α-synuclein (αSN) aggregates	(αSN aggr inh) 10–100 μM	[34]
*Ampelopsis grossedentata*	Dihydromyricetin	Neuroprotective activity and inhibition of αSN fibril formation	(αSN aggr inh) 50–100 μM	[35]

**Table 2:** Natural compounds with potential use in the therapy of metabolic syndrome and/or with additional effects for the reduction of cardiovascular risk.

Source	Bioactive Compounds	Effects	Main Activities	Ref.
*Acalypha fluticosa*	2-methyl-5,7-dihydroxychromone 5-*O*-b-d-glucopyranosid, acalyphin, apigenin, and Kaempferol 3-*O*-rutinoside and an acetylated derivate of chromone glucoside	Agonism of PPARα and PPARγ; anti-inflammatory properties	(PPARα act., FI) 1.16–2.25 at 50 μM	[70]
*Talisia nervosa* Radlk	(–)-catechin, methyl gallate, ethyl gallate, and β-d-glucopyranose,1,4,6-tris(3,4,5-trihydroxybenzoate)	Agonism of PPARα, PPARγ and liver X receptor (LXR); reduction of NO production	(PPARγ act., FI) 1.85–3.02 at 50 μM	[71]
*Penicillium chrysogenum* J08NF-4	A new bile acid trifluoroacetate	Agonism of PPARγ, anti-inflammatory properties	(PPARγ act., FI) 2.0 at 50 μM	[72]
*Cyanobium* sp. LEGE 07,175 and *Nodosilinea* sp. LEGE 06001	13^2^-hydroxy-pheopytin a and 13^2^-hydroxy-pheofarnesin a	Increase of PPARγ mRNA expression	(Lipid-reducing Activity, EC_50_) 8.9–15.5 μM	[73]
*Penicillium expansum* Y32	Communesin A, B, I, fumiquinazoline Q, protuboxepin A, B, E, and others	Mitigation of bradycardia, vasculo-genetic effect	(Acid sphingomyelinase mitigation) 20–100 μM	[58]
Lichens	Thirty-seven secondary metabolites and semisynthetic derivates	Anti-AGE activity, vasodilation	(Pentosidine-like AGEs formation, IC_50_) 0.08–0.70 mM	[74]
*Schisandra chinensis*	Acidic polysaccharide (SCAP)	Anti-diabetic and anti-apoptotic role	(H_2_O_2_-induced apoptosis inh) 15.6–62.5 μM	[75]
*Sesbania grandiflora*	Quercetin, kaempferol, vomifoliol, loliolide and others	Inhibition of α-amylase and α-glucosidase; antioxidant activity	(α-Glucosidase inh, IC_50_) 17.45–388.48 μM	[76]
*Cassia bakeriana* craib	Kaempeferol-3-*O*-rhamnoside and kaempferol	Inhibition of α-amylase and antioxidant activity	(α-Glucosidase inh, IC_50_) 0.36–0.61 mg/mL	[77]
*Ocimum campechianum* Mill.	Methyl rosmarinate, rosmarinic acid, 5-demethyl nobiletin, 5-demethyl sinensetin, luteolin	Inhibition of α-glucosidase, antihyperglycemic action	(α-Glucosidase inh) 12.86–82.77% at 0.75 mM	[78]
*Aspergillus terreus* MC751	Butyrolactone I and II, three acetylated derivates of butyrolactone I	Inhibition of α-glucosidase, antioxidant activity	(α-Glucosidase inh, IC_50_) 52.17–175.18 μM	[79]
*Ganoderma australe*	Stella-steroid	Inhibition of α-glucosidase and Dipeptidyl peptidase 4 (DPP-4)	(α-Glucosidase inh, IC_50_) 314.54 μM	[80]
*Aspergillus sydowii*	Asperentin B	Inhibition of Protein-tyrosine phosphatase 1B (PTP1B)	(PTP1B inh, IC_50_) 2.05 μM	[81]
*Moringa oleifera*	Two sulfur-contained compounds	Anti-adipogenic activity	(Lipid accumulation, inh, IC_50_) 29.6 μM	[82]
*Allium sativum* L.	Three eugenol diglycosides and three β-carboline alkaloids	Inhibition of adipogenesis and lipid accumulation	(Lipid accumulation, inh) active at 20 μM	[83]
*Curcuma amada*	Two natural labdane diterpenes and one drimane sesquiterpene	Inhibition of lipase and α-glucosidase	(Lipase inh, IC_50_) 6.1–665.9 μM	[84]
*Magnolia* spp.	Honokiol	Inhibition of Histone deacetylase (HDAC)- mediated cystathionine γ-lyase degradation	(HDAC6 inh) active at 5 μM	[85]
*Panax* spp. *(Ginseng)*	Ginsenoside K	Promotion of macrophage and foam cell apoptosis	(Reduction of foam cell formation) 1.25 μg/mL	[86]
*Unspecified*	Lupeol	Promotion of macrophage development into the M2 anti-inflammatory phenotype	(Proinflammatory cytokine secretion, inh) active at 50 μM	[87]
*Tripterygium Wilfordi*	Cerastrol	Action as leptin sensitizer	(Leptin sensitization) active at 150 μg/kg	[88]
